# Lignans and breast cancer risk in pre- and post-menopausal women: meta-analyses of observational studies

**DOI:** 10.1038/sj.bjc.6605003

**Published:** 2009-03-31

**Authors:** L S Velentzis, M M Cantwell, C Cardwell, M R Keshtgar, A J Leathem, J V Woodside

**Affiliations:** 1 Department of Surgery, Breast Cancer Research Group, University College London, Charles Bell House, 67-73 Riding House St, London W1W 7EJ, UK; 2 Cancer Epidemiology and Prevention Research Group, Centre for Public Health, Queen's University Belfast, Mulhouse Building, Grosvenor Road, Belfast BT12 6BJ, UK; 3 Department of Surgery, Royal Free Hospital, Pond St, London NW3 2QG, UK; 4 Nutrition and Metabolism Group; Centre for Public Health, Queen's University Belfast, Mulhouse Building, Grosvenor Road, Belfast BT12 6BJ, UK

**Keywords:** plant lignans, enterolignans, breast cancer risk

## Abstract

Phyto-oestrogens are plant compounds structurally similar to oestradiol, which have been proposed to have protective effects against breast cancer. The main class of phyto-oestrogens in the Western diet is lignans. Literature reports on the effect of lignans in breast cancer risk have been conflicting. We performed three separate meta-analyses to examine the relationships between (i) plant lignan intake, (ii) enterolignan exposure and (iii) blood enterolactone levels and breast cancer risk. Medline, BIOSIS and EMBASE databases were searched for publications up to 30 September 2008, and 23 studies were included in the random effects meta-analyses. Overall, there was little association between high plant lignan intake and breast cancer risk (11 studies, combined odds ratio (OR): 0.93, 95% confidence interval (95% CI): 0.83–1.03, *P*=0.15), but this association was subjected to marked heterogeneity (*I*^2^=44%). Restricting the analysis to post-menopausal women, high levels of plant lignan intake were associated with reduced breast cancer risk (7 studies, combined OR: 0.85, 95% CI: 0.78, 0.93, *P*<0.001) and heterogeneity was markedly reduced (*I*^2^=0%). High enterolignan exposure was also associated with breast cancer (5 studies, combined OR: 0.73, 95% CI: 0.57, 0.92, *P*=0.009) but, again, there was marked heterogeneity (*I*^2^=63%). No association was found with blood enterolactone levels (combined OR: 0.82, 95% CI: 0.59–1.14, *P*=0.24). In conclusion, plant lignans may be associated with a small reduction in post-menopausal breast cancer risk, but further studies are required to confirm these results.

High levels of endogenous circulating oestrogens (Hankinson and Eliassen, 2007) and use of exogenous oestrogens (Beral, 2003) have both been associated with increased breast cancer risk. Isoflavones and lignans are plant compounds structurally similar to 17*β*-oestrodiol known as phyto-oestrogens, capable of oestrogen receptor binding (Kuiper *et al*, 1998; Mueller *et al*, 2004). Isoflavones are mostly found in soybean products, which are a staple of the Asian diet, whereas lignans are the principal group of phyto-oestrogens in Western diets. Lignans are more widespread in foods than isoflavones and are present in grain cereals, vegetables, seeds, tea and coffee (Mazur, 1998a; Mazur *et al*, 1998b). Microflora in the colon (Setchell *et al*, 1981) convert plant lignans into enterolignans, which are detectable in blood and urine. Their levels have been correlated with the amount of plant lignans ingested (Nesbitt *et al*, 1999).

In a recent meta-analysis, an inverse dose-response relationship was shown between breast cancer risk and soy-food intake in Asian, but not in Western women (Wu *et al*, 2008). Lignans have been shown to exhibit anti-carcinogenic properties (Wang *et al*, 1994; Prasad 2000; Bergman Jungeström *et al*, 2007), and it is hypothesised that exposure to high levels may be associated with a reduction in breast cancer risk. However, results from a number of studies in Western populations have been variable. The aim of our systematic review was to establish whether an association exists between lignan exposure and breast cancer risk, and to quantify the association through meta-analyses to inform evidence-based dietary guidelines.

## Materials and methods

A systematic search of Ovid Medline (US National Library of Medicine, Bethesda, MD, USA), BIOSIS (Thompson Reuters, NY, USA) and EMBASE (Reed Elsevier PLC, Amsterdam, The Netherlands) databases for relevant studies published up to and including the date, 30 September 2008 was carried out. Relevant studies included at least one keyword or Medical Subject Heading from each of the following; (i) plant lignans (matairesinol, secoisolarisiresinol, pinoresinol and lariciresinol), (ii) enterolignans (enterolactone and enterodiol) and (iii) breast cancer. The search strategy excluded reviews, animal and cell culture studies but did not impose any language restrictions.

Abstracts and full texts, where required, were independently screened by two investigators to establish the suitability for inclusion. Studies had to be of case–control or cohort design, evaluating the risk of invasive breast cancer in relation to lignan exposure and reporting odds ratios (ORs) or relative risks, as well as 95% confidence intervals (95% CIs). Cited references were also reviewed for any studies that may have been missed in the database searches.

Eligible publications were then assessed independently by three reviewers. A structured form was used to extract information about the study, subjects’ characteristics including menopausal status, confounding factors and results. Wherever multiple publications of the same study were available, the paper with the most complete set of data was chosen.

Studies were then categorised as those: (i) assessing total plant lignan intake or intake of individual plant lignans if the total was not measured; (ii) investigating exposure to enterolignans (enterolactone and enterodiol) by using values produced from food by *in vitro* fermentation models; and (iii) examining enterolactone levels in the blood (either plasma or serum). The blood levels of enterodiol were measured in a small number of studies (Piller *et al*, 2006b; Verheus *et al*, 2007; Ward *et al*, 2008) and were, therefore, not considered for analysis.

Separate meta-analyses were performed for each group of studies described in the Methods section using adjusted ORs or relative risks for the highest *vs* the lowest categories of exposure. If different levels of adjustment had been carried out, the results from the most fully adjusted model were used.

Random effects models were used to calculate pooled estimates, as we anticipated heterogeneity between observational studies (DerSimonian and Laird, 1986). Study-specific weights in the random effects model were calculated and scaled to percentages. The *I*^2^-statistic was used to test for heterogeneity (Higgins *et al*, 2003). Publication or selection bias was investigated by checking for asymmetry in funnel plots (Egger *et al*, 1997).

Analysis was repeated and sub-divided by menopausal status (pre- and post-menopausal). Statistical analyses were performed using the STATA version 9.2 software (Stata Corporation 2005, College Station, TX, USA).

## Results

Following screening of abstracts and full texts and grouping into categories, 27 of the 33 articles identified were selected for data extraction. Multiple publications were identified for a number of studies. Four articles (Grace *et al*, 2004; McCann *et al*, 2006; Thanos *et al*, 2006; Piller *et al*, 2006b) were excluded, as they were based on smaller subgroup analysis of their respective larger studies. The format of certain results prevented their use, but were provided by the authors in a suitable form and therefore included in this study. Overall, 23 publications were used, providing data for 6 cohort, 6 nested case–control and 10 case–control studies. Each article contributed data to one or more meta-analyses resulting in 12 articles on plant lignan intake (see Table 1), 5 on enterolignan exposure (see Table 2) and 9 on blood enterolactone levels (see Table 3). Details of the adjustments made in each study (the most fully adjusted model was used in the meta-analysis) are shown in Tables 1, 2, 3.

**Figure 1:**
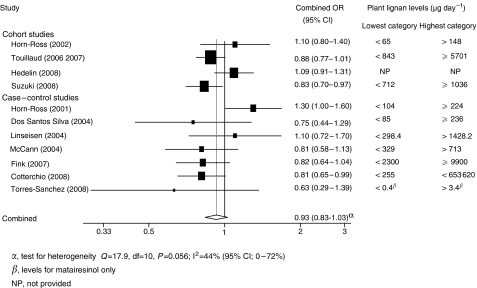
Forest plot of highest *vs* lowest plant lignan intake and breast cancer risk.

**Figure 2:**
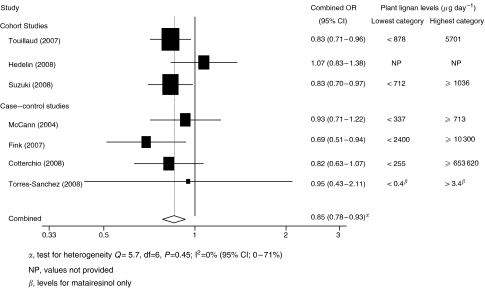
Forest plot of highest *vs* lowest plant lignan intake and breast cancer risk in post-menopausal women.

There was no association between plant lignan intake and risk when 11 studies were combined, although there was a slight protective effect. The risk in the highest intake group was 0.93 times (95% CI: 0.83–1.03, *P*=0.15) that of the lowest intake group (see Figure 1). When studies were analysed by menopausal status, a statistically significant reduction in risk was seen with the highest intake category of plant lignans *vs* the lowest intake in post-menopausal women (7 studies, combined OR: 0.85, 95% CI: 0.78, 0.93, *P*<0.001), with little sign of between-study heterogeneity (*I*^2^=0%, 95% CI: 0, 71, *P*=0.46) (see Figure 2). The same effect was not observed in pre-menopausal women (7 studies, combined OR: 0.97, 95% CI: 0.82, 1.15, *P*=0.73). The funnel plot of studies examining plant lignan intake and overall breast cancer risk showed symmetry, suggesting a lack of publication bias.

**Figure 3:**
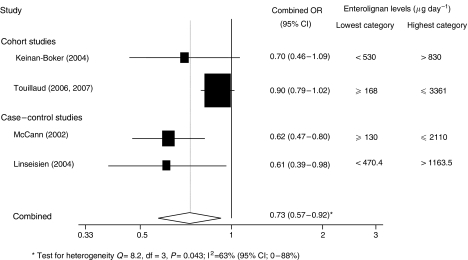
Forest plot of highest *vs* lowest level of enterolignan exposure and breast cancer risk.

There was a statistically significant inverse association between enterolignan exposure and overall risk (combined OR: 0.73, 95% CI: 0.57, 0.92, *P*=0.009) (Figure 3), although there was marked heterogeneity (*I*^2^=63%, 95% CI: 0.0, 88, *P*=0.04), but there was no association between exposure and risk by menopausal status (pre-menopausal breast cancer risk: 3 studies, combined OR: 0.67, 95% CI: 0.44–1.02, *P*=0.06; post-menopausal: 2 studies, combined OR: 0.85, 95% CI: 0.72–1.01, *P*=0.06).

**Figure 4:**
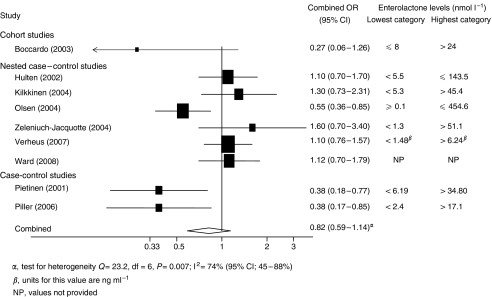
Forest plot of highest *vs* lowest enterolactone levels in blood and breast cancer risk.

There was no association between blood enterolactone and breast cancer risk (combined OR: 0.82, 95% CI: 0.59–1.14, *P*=0.24) (Figure 4). Results of analysis by menopausal status were similar for both pre-menopausal women (5 studies, combined OR: 0.85, 95% CI: 0.45–1.59, *P*=0.61) and post-menopausal women (6 studies, combined OR: 0.86, 95% CI: 0.66, 1.14, *P*=0.28).

## Discussion

This is the first systematic review and meta-analysis of exposure to lignans and breast cancer risk based on studies using dietary assessments and serum measurements. Although exposure can be assessed by urine analysis, few studies have used this methodology and therefore, these were not included (Ingram *et al*, 1997; den Tonkelaar *et al*, 2001; Dai *et al*, 2002). The results show that there was no association between plant lignan intake and overall risk, and this association was subjected to marked heterogeneity. However in post-menopausal women, there is a small but significant reduction in risk and a reduction in heterogeneity. A significantly decreased risk with increasing enterolignan exposure was also found. However, there was significant heterogeneity between studies making it difficult to draw clear conclusions, and the effect did not persist when analyses were stratified by menopausal status, although the number of studies included in these stratified analyses was very small. Finally, there was no association between enterolactone concentrations in blood and overall risk, or when analysis was stratified by menopausal status.

The protective action of plant lignans against breast cancer in post-menopausal, but not in pre-menopausal women, would suggest that lignan activity has a physiologic effect only at low oestradiol levels. One of the mechanisms of action may be greater sex hormone-binding globulin production and binding of free oestradiol (Adlercreutz *et al*, 1989, 1992; Zeleniuch-Jacquotte *et al*, 2004; Low *et al*, 2007). Binding of type II nuclear oestrogen receptor (Adlercreutz *et al*, 1992; Adlercreutz, 2007) and altering oestrogen synthesis within the breast cells and extragonadal sites, such as the adipose tissue, are other possible mechanisms (Adlercreutz *et al*, 1993; Saarinen *et al*, 2007). Enterolactone has been shown to decrease local oestrogen production by inhibiting 17-hydroxysteroid dehydrogenase type I and aromatase (Wang *et al*, 1994; Brooks and Thompson, 2005).

The apparent protective effect of dietary plant lignans in post-menopausal women is not supported by the findings from the meta-analysis of studies that measured the enterolactone levels in their blood. It would be expected that women consuming larger amounts of plant lignans would have a higher circulating concentration of enterolactone. There are a number of possible reasons for this disparity. Dietary intake of plant lignans was assessed on the basis of the subjects’ self-reported dietary intake ranging from 6 months before study entry (Hedelin *et al*, 2008) to 3 years before breast cancer diagnosis, (dos Santos Silva *et al*, 2004) and thus, it reflects long-term intake. Enterolactone concentration that is measured in a single blood sample may be more indicative of recent dietary habits. There may also be a significant intra-individual variation in serum response to dietary intake of plant lignans (Hausner *et al*, 2004). For example, blood levels of enterolactone can be modulated by age, smoking, frequency of defecation, weight–obesity–body mass index and regular alcohol intake (Kilkkinen *et al*, 2001, 2002; Horner *et al*, 2002; Milder *et al*, 2007), and these factors could potentially differ by menopausal status (in particular, age and body mass index). As bacterial enzymes are involved in lignan metabolism, the use of antibiotics has also been shown to affect enterolactone serum concentration (Kilkkinen *et al*, 2002); antibiotic use was generally not controlled for in these studies.

It is also possible that the protective effect is caused directly by the plant lignans or chemicals within the metabolic pathway other than enterolactone, or even by a synergistic effect between plant lignans and enterolignans. However, other food constituents found to be associated with plant lignans may exert the effect. For example, *α*-linoleic acid, which is also thought to have anti-cancer effects (Thompson, 2003; Bougnoux and Chajes, 2003, p. 232), is found in very high levels in flaxseed, the richest source of plant lignans (Thompson *et al*, 1991).

Determining plant lignan intake has various limitations, which could lead to an over- or under-estimation of food content. Some food composition databases are incomplete in terms of not containing values for the more recently discovered plant lignans (e.g., medioresinol) or for the whole range of foods consumed by the study population. In addition, there are various analytical methods for determining food values ; hence, databases compiled from published values determined by different methodologies may contain inherent errors. It has also been shown that the amount of lignans in food can differ according to crop variety, location, year of harvest and processing (Thompson *et al*, 1997; Kuijsten *et al*, 2005). Dietary measurement error associated with FFQs (food frequency questionnaires) is also possible. FFQs that were used varied in length, ranging from 67 to 208 items. Only one study validated its FFQ specifically for plant lignan assessment (Torres-Sanchez *et al*, 2008), although a UK study used the combination of an FFQ and 24-h recalls to group participants into quartiles of intake (dos Santos Silva *et al*, 2004). In addition, the possibility of residual confounding cannot be ruled out.

Consumption of soy food, rich in isoflavones, has been shown to reduce breast cancer risk in Asian women but not in Western women (Wu *et al*, 2008), suggesting that ethnicity may play a role in this effect. It is not known whether there are differential physiologic effects of lignans in people of different races, although there is some evidence of variation in the urinary excretion of lignans between white, African American and Latino women (Horn-Ross *et al*, 1997). Of the 23 articles used for the meta-analyses, only 3 American studies provided complete data with regard to ethnicity (Horn-Ross *et al*, 2001, 2002; McCann *et al*, 2004); hence, it was impossible perform sub-analyses for examining this.

In summary, the meta-analyses presented in this study, indicate that plant lignans and enterolignans are unlikely to significantly protect all women against breast cancer development. However, our results suggest that high plant lignan intake is associated with a 15% decreased risk in post-menopausal women, which is a small reduction that could be due to residual confounding. If real, the reason for the selective effect is not clear. Additional studies of the effect of lignan exposure on post-menopausal breast cancer risk are needed to confirm these findings before reassessing the current dietary guidelines.

## Figures and Tables

**Table 1:** Characteristics of studies included in the review of plant lignans and breast cancer risk

**First author/ (year)/ country**	**Parent study**	**Study design (follow-up)**	**Cases**	**Controls/ cohort size**	**Menopausal status**	**Lignans measured**	**Dietary assessment**	**Adjusted confounders**
Horn-Ross *et al* (2002) United States	California Teachers Study	Prospective cohort (222 249 person-years; 2 years^**^)	711	111 526	Pre-M and Post-M	M, S	Self-reported 113-item FFQ	Age at 1st birth and menarche, BMI, daily caloric intake, ethnicity, family history, menopausal status, nulliparity, physical activity
Touillaud *et al* (2006) France	E3N Study	Prospective cohort (117 652 person-years; 4.2 years^*^)	402	26 868	Pre-M	M, S, P, L	Self-reported 208-item FFQ	Age at 1st birth and at menarche, alcohol, BBD, BMI, education, family history, energy, geographic area, height, OC, parity
Touillaud *et al* (2007) France	E3N Study	Prospective cohort (383 425 person-years; 7.7 years^*^)	1469	58 049	Post-M	M, S, P, L	Self-reported 208-item FFQ	Age at 1st birth, at menarche menopause, alcohol, BBD, BMI, energy, family history, height, HRT, OC, parity, smoking
Hedelin *et al* (2008) Sweden	SWLH cohort	Prospective cohort (13 years)	1014	1014	Pre-M and Post-M	M, S, P, L, Sy, Med	Self-reported 80-item FFQ	Age at menarche and 1st pregnancy, alcohol, BMI, energy, family history, OC, parity, saturated fat
Suzuki *et al* (2008) Sweden	SMC Study	Prospective cohort (430 339 person-years; 8.3 years^**^)	1284	51 823	Post-M	M, S, P, L	Self-reported 67-item FFQ (1987), 93-item FFQ (1997)	Age at 1st birth, menarche and menopause, alcohol, BBD, BMI, education, energy, family history, height, HRT, OC, parity
Horn-Ross *et al* (2001) United States	Bay Area Breast Cancer Study	Population-based case–control	1272	1610	Pre-M and Post-M	M, S	Self-reported 94-item FFQ	Age, age at menarche, BBD, BMI, daily caloric intake, education, family history, HRT, lactation, menopausal status, parity, race
dos Santos Silva *et al* (2004) United Kingdom		Case–control (GP's patient lists)	240	477	Pre-M and Post-M	M, S	Interviewed 207-item FFQ	Age at 1st birth and at menarche, education, family history, lactation, menopausal status, parity
Linseisen *et al* (2004) Germany		Population-based case–control	278	666	Pre-M	M, S	Self-reported 176-item FFQ	Alcohol, BMI, education, energy, family history, lactation, parity
McCann *et al* (2004) United States	WEB Study	Population-based case–control	1122	2036	Pre-M and Post-M	M, S	Self-reported 98-item FFQ	Age, age 1st birth, at menarche and menopause, BBD, BMI, education, energy, age at menopause, parity, race, smoking
Fink *et al* (2007) United States	LIBCSP Study	Population-based case–control	1434	1404	Pre-M and Post-M	M, S	Self-reported 94-item FFQ	Age and energy
Cotterchio *et al* (2008) Canada	Ontario Women's Diet and Health Study	Population-based case–control	3063	3370	Pre-M and Post-M	M, S, P, L	Self-reported 178-item FFQ	Age, age at 1st live birth, BBD, dietary fibre intake, family history, HRT
Torres-Sanchez *et al* (2008) Mexico		Hospital based case–control	141	141	Pre-M and Post-M	M, S, P, L	Interviewed 100-item FFQ	Age, energy, lifetime lactation, menopause status

BBD=benign breast disease; BMI=body mass index; E3N=French Component of the European Prospective Investigation into Diet and Cancer (EPIC) Study; FFQ=food frequency questionnaire; GP=general practitioner; HRT=hormone replacement therapy; L=lariciresinol; LIBCSP=Long Island Breast Cancer Study Project; M=matairesinol; Med=medioresinol; OC=oral contraceptive; P=pinoresinol; Peri-M=peri-menopausal; Pre-M=pre-menopausal; Post-M=post-menopausal; S=secoisolariciresinol; SMC=Swedish Mammography Cohort; SWLH=Scandinavian Women's Lifestyle and Health Cohort; Sy=syringaresinol; WEB=Western New York Exposure and Breast Cancer Study.

^*^Median follow-up; ^**^Mean follow-up.

**Table 2:** Characteristics of studies included in the review of mammalian enterolignans (enterolactone and enterodiol) and breast cancer risk

**First author/ (year)/ country**	**Parent study**	**Study design (median follow-up)**	**Cases**	**Controls/ cohort size**	**Menopausal status**	**Diet assessment**	**Adjusted confounders**
Keinan-Boker *et al* (2004) The Netherlands	Prospect- EPIC	Prospective Cohort (5.2 years)	280	80 215	Pre-M, Peri-M and Post-M combined	Self-reported 178-item FFQ	Age at 1st birth and study entry, education, energy, height, HRT, marital status, OC, parity, physical activity, weight
Touillaud *et al* (2006) France	E3N Study	Prospective Cohort (4.2 years)	402	117 652	Pre-M	Self-reported 208-item FFQ	Age at 1st birth and menarche, alcohol, BBD, BMI, education, energy, family history, geographic area, height, OC, parity
Touillaud *et al* (2007) France	E3N Study	Prospective cohort (7.7 years)	1469	383 425	Post-M	Self-reported 208-item FFQ	Age at 1st birth, at menarche and menopause, alcohol, BBD, BMI, energy, family history, geographic area, height, HRT, OC, parity, smoking
McCann *et al* (2002) United States	WEB Study	Population-based case–control	301 439	316 494	Pre-M Post-M	FFQ	Age at menarche, BBD, BMI, education, energy, family history, parity; further adjusted for age at menopause
Linseisen *et al* (2004) Germany		Population-based case–control	278	666	Pre-M	Self-reported 176-item FFQ	Alcohol, BMI, breast-feeding, education, energy, family history, parity; controls matched by exact age to cases

BBD=benign breast disease; BMI=body mass index; E3N=French Component of the European Prospective Investigation into Diet and Cancer (EPIC) Study; FFQ=food frequency questionnaire; HRT=hormone replacement therapy; OC=oral contraceptive use; Peri-M=Peri-menopausal; Pre-M=pre-menopausal; Post-M=post-menopausal; Prospect-EPIC=Dutch Cohort of EPIC Study; WEB=Western New York Exposure and Breast Cancer Study.

**Table 3:** Characteristics of studies included in the review of enterolactone exposure as measured in blood and breast cancer risk

**First author/ (year)/ country**	**Parent study**	**Design (follow-up)**	**Cases**	**Controls/ cohort size**	**Method**	**Menopausal status**	**Mean ENL cases (nmol/l)**	**Mean ENL controls/cohort (nmol/l)**	**Adjusted confounders**
Boccardo *et al* (2003) Italy		Prospective cohort (6.5 years after cyst aspiration)	18	383	TR-FIA	Pre-M and Post-M	14.7	19.6	Age, cyst type and family history
Hultén *et al* (2002) Sweden	VIP, MONIKA and MSP studies	Nested case–referent	248	492	TR-FIA	Pre-M and Post-M	26.8 VIP and MONIKA 19.3 MSP	22.9 VIP and MONIKA 20.4 MSP	BMI, menopausal status, smoking
Kilkkinen *et al* (2004) Finland	Cross-sectional population surveys	Nested case–control	206	215	TR-FIA	Pre-M and Post-M	25.2	24.0	None
Olsen *et al* (2004) Denmark	Diet, Cancer and Health Study	Nested case–control	381	381	TR-FIA	Post-M	Not provided	Not provided	Age, HRT (through matching of controls)
Zeleniuch-Jacquotte *et al* (2004) United States	NYU Women's Health Study	Nested case–control	417	417	TR-FIA	Pre-M Post-M	18.3 18.6	15.1 18.9	Age at 1st live birth and menarche, ln(BMI), family history, ln(height), nulliparity
Verheus *et al* (2007) The Netherlands	Prospect-EPIC	Nested case–control	383	383	LC/MS	Pre-M/Peri-M Post-M	2.98 (ng/ml) 2.71 (ng/ml)	2.66(ng/ml) 2.65 (ng/ml)	Age at menarche and family history (Pre-M) Crude OR (Post-M).
Ward *et al* (2008) United Kingdom	EPIC-Norfolk	Nested case–control (9.5 years; 11 261 person-years)	219	891	LC/MS	All	5.83 (ng/ml)^*^	5.00 (ng/ml)^*^	Age, age at menarche, breast-feeding, energy, family history, fat, HRT, OC, menopausal status, parity, social class, weight
Pietinen *et al* (2001) Finland	Kuopio Breast Cancer Study	Population-based case–control	194	208	TR-FIA	Pre-M Post-M	16.6 21.2	20.7 28.9	Age at 1st birth and at menarche, alcohol, area, BBD, BMI, education, family history, HRT, OC, physical activity, smoking, waist to hip ratio
Piller *et al* (2006a) Germany		Population-based case–control	192	231	TR-FIA	Pre-M	11.6	12.2	Age at menarche, alcohol, BMI, breast-feeding, day of analysis, education, family history, OC, parity, time difference between surgery and blood sampling day

BBD=benign breast disease; BMI=body mass index; ENL=enterolactone; HRT=hormony replacement therapy; LC=liquid chromatography; MONIKA=Monitoring of Trends and Cardiovascular Disease Study; MS=mass spectrometry; MSP=Mammary Screening Project; NYU=New York University; OC=oral contraceptive; Peri-M=peri-menopausal; Pre-M=pre-menopausal; Post-M=post-menopausal; Prospect-EPIC=Dutch Cohort of the European Prospective Investigation into Diet and Cancer (EPIC) Study; TR-FIA=time-resolved fluoroimmunoassay; VIP=Västerbotten Intervention Project.

^*^Median values. Mean values not provided.
